# Assessment of the piroxicam‐incited model of synchronized colitis in T‐cell receptor alpha chain‐deficient mice

**DOI:** 10.1002/ame2.12456

**Published:** 2024-07-11

**Authors:** Maximo E. Lange, Danisa M. Bescucci, Valerie F. Boras, Tony Montina, G. Douglas Inglis

**Affiliations:** ^1^ Lethbridge Research and Development Centre Agriculture and Agri‐Food Canada Lethbridge Alberta Canada; ^2^ Alberta Health Services Chinook Regional Hospital Lethbridge Alberta Canada; ^3^ Department of Chemistry and Biochemistry University of Lethbridge Lethbridge Alberta Canada; ^4^ Southern Alberta Genome Sciences Centre University of Lethbridge Lethbridge Alberta Canada

**Keywords:** colon, dexamethasone, histology, induction, inflammation, knockout

## Abstract

**Background:**

A multitude of mouse models are utilized to emulate and study intestinal inflammation. T‐cell receptor alpha chain (TCRα)‐deficient mice are used as a model of spontaneous colitis that has similarities with human ulcerative colitis. However, colitis is triggered late in the life of the mouse (age: 4–5 months), and inflammation does not develop at the same time in different mice. A previously conducted study reported that the administration of the drug piroxicam triggered predictable and early colitis in TCRα‐deficient mice at the age of 6–8 weeks. However, a detailed characterization of ensuing inflammation was not provided.

**Methods:**

We conducted an in‐depth examination of piroxicam‐triggered colitis in TCRα‐deficient mice, with emphasis on spatial histopathologic changes and analysis of expression of inflammatory markers. Furthermore, we tested amelioration of colitis with dexamethasone.

**Results:**

We confirmed that piroxicam induced a time‐prescribed colitis and did so in the proximal colon as well as the cecum of TCRα‐deficient mice. Piroxicam administration was observed to induce epithelial hyperplasia, goblet cell loss, and leukocyte infiltration with occasional ulceration. A Swiss roll technique was used to examine the colon and cecum in its entirety. Importantly, we observed that inflammation was multifocal segmental, with areas of tissue damage in between healthy tissue. In addition, we observed variability in the severity of inflammation among replicate animals and treatments, and that the administration of dexamethasone only partially ameliorated inflammation in the proximal colon.

**Conclusions:**

Piroxicam consistently induced multifocal segmental colitis in the proximal colon and cecum, although the degree of inflammation was reduced in the latter. Importantly, spatial variability in inflammation in the large intestine and the inter‐replicate variation in the severity of inflammation must be taken into consideration when utilizing this murine model of synchronized colitis.

## Graphical abstract

We conducted an in depth examination of piroxicam‐triggered colitis in T cell receptor α‐deficient mice. Piroxicam induces a prescribed inflammation with occasional presence of ulcers in 6–8 week old mice in the colon and to a lesser degree in the cecum. Administration of dexamethasone only partially ameliorates inflammation.

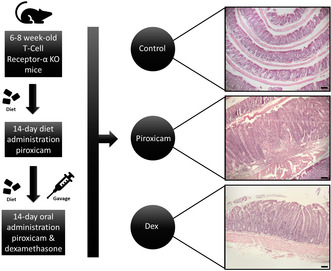

## 1 INTRODUCTION

Controlled intestinal inflammation is a process that is essential to regulate the elimination of pathogens, balance immunological function, and coordinate the interaction of the host with commensal bacteria and food particles. However, a loss in the delicate balance that regulates the immune response can lead to uncontrolled intestinal inflammation. This can result in clinical manifestations that are accompanied with disruption of the gut epithelial barrier and dysregulation of homeostasis, eventually developing into disease.^1^ A large variety of etiologies can trigger acute inflammation, including infection by pathogens, exposure to adverse environmental conditions, and/or host genetic factors.^1^, ^2^, ^3^ Given the complexity of elements at play in an inflammatory response, live models are used to elucidate mechanisms and to develop effective mitigations. As such, a large diversity of murine models of intestinal inflammation are used,^4^ including the T‐cell receptor alpha chain (TCRα)‐deficient mouse model. This model was developed in 1993 by Mombaerts et al.,^5^ and it is characterized by the spontaneous development of chronic inflammation in the large intestine that shares similarities with ulcerative colitis. TCRα‐deficient mice spontaneously develop colitis at the age of 4–5 months, with potential mortality starting at the age of 6 months, and it has been used as an intestinal inflammation model in multiple reports.^6^, ^7^, ^8^, ^9^, ^10^ Colitis in the TCRα‐deficient mouse model is mediated by Th2 cells, and it is characterized by crypt hyperplasia accompanied by elongation of crypts and loss of goblet cells.^5^ The spontaneous colitis characteristic of this model has its challenges, given that mice are disease free for a period of 4–5 months, and colitis develops sporadically, which greatly complicates the standardization of experimental time lines. To address this challenge, Nishyiori et al.^11^ induced colitis in 6‐week‐old TCRα‐deficient mice by orally administering the nonsteroidal anti‐inflammatory drug (NSAID), piroxicam, daily for 14 days. The synchronization of colitis after the administration of piroxicam enables the practical use of this model (e.g., to evaluate mitigations). However, an in‐depth histopathological analysis of the inflammation of the entire colon and cecum has not been completed. As such, similarities and differences with the spontaneous model have not been fully described.

As a prelude to using the TCRα‐deficient piroxicam‐induction model to develop and test novel mitigations for colitis, we completed an in‐depth histological analysis in conjunction with an examination of inflammatory markers. Emphasis was placed on determining the spatial heterogeneity of colitis. Moreover, we assessed the impact of the glucocorticoid anti‐inflammatory dexamethasone on disease. We observed that piroxicam induced a multifocal segmental inflammation with epithelial hyperplasia, loss of goblet cells, and the occasional presence of ulcers that were primarily located in the proximal colon. Besides colitis in the proximal colon, inflammation occurred in the cecum. In contrast to Nishyiori et al.,^11^ we found that the administration of dexamethasone at a dose of 3 mg/kg did not consistently ameliorate histopathologic changes of inflammation; in some mice, dexamethasone effectively mitigated inflammation (17%), whereas in other mice it did not (83%). However, the administration of dexamethasone consistently reduced the expression of inflammatory genes in mice administered piroxicam. Crucially, the spatial heterogeneity of inflammation that occurred in the large intestine of TCR‐deficient mice after the administration of piroxicam necessitates that this be considered when using this model of synchronized colitis.

## 2 METHODS

### 2.1 Animals and husbandry

Homozygous TCRα‐deficient (TCRα^−/−^) male mice (B6.129S2‐*Tcratm1Mom*/J) were purchased from Jackson Laboratory (ME). After being transported to LeRDC, 5‐week‐old mice were housed in Tecniplast Green Line GM500 Sealsafe Plus individually ventilated cages (IVC) (Tecniplast, Toronto, ON, Canada) situated in a single‐sided Sealsafe Plus rack (Tecniplast) attached to a Smart Flow (Tecniplast) high‐efficiency particulate air (HEPA) filtered air handling unit. Mice were provided a gamma‐irradiated AIN‐93G mouse diet (Dyets Inc., Bethlehem, PA, USA) and allowed to drink and eat ad libitum. Mice were maintained on a 12‐h light/dark cycle, and they were allowed to acclimatize for a 1‐week period before commencement of the experiment.

### 2.2 Experimental design and treatment administration

The experiment was designed as a single‐factor experiment with three treatments and six replicate mice per treatment. The treatments were (1) control, (2) piroxicam, and (3) piroxicam + dexamethasone (DexP). Piroxicam in diet (200 ppm w/w; Alfa‐Aesar, Ottawa, ON, Canada) was continuously administered to mice that were randomly assigned to treatments two and three, whereas mice assigned to treatment one were provided diet without piroxicam. Control and piroxicam treatment mice were orally gavaged with 100 μL of sterile water containing 0.5% (w/v) β‐cyclodextrin (BCD; Sigma‐Aldrich, St. Louis, MO, USA), whereas DexP treatment mice were gavaged with dexamethasone (3 mg/kg; Alfa‐Aesar, Mississauga, ON, Canada) in sterile water containing BCD. BCD was included to solubilize dexamethasone in water. Each animal was gavaged once every morning using a 22‐G gavage needle and a mouse restraint cone (DecapiCone, Braintree Scientific, Braintree, MA, USA) for 14 consecutive days.

### 2.3 Animal health status and tissue collection

The health status of mice was monitored at least twice daily. On the morning of experiment Day 15, mice were weighed, anesthetized with isoflurane, and humanely euthanized under general anesthesia by cervical dislocation. To obtain tissues, a mid‐laparotomy was performed. The cecum, proximal colon, and distal colon were aseptically removed. The colon was weighed, and total colonic length was measured. The exterior of the intestine was examined for evidence of inflammation. The intestine was then incised longitudinally, the mucosa was carefully examined and photo‐documented, and evidence of gross pathologic changes was recorded (e.g., ulceration). Within 5 min of euthanization, subsamples of tissues from all three intestinal locations were placed in RNAlater stabilization solution (Thermo Fisher Scientific Inc., Waltham, MA, USA) for messenger RNA (mRNA) quantification. Remaining intestinal tissues were sampled using a Swiss roll technique^12^ placed in TrueFlow Macrosette cassettes (Tissue Path; Thermo Fisher Scientific Inc.) and stored in 10% neutral buffer formalin (Leica Biosystems, Concord, ON, Canada) for histopathologic analysis.

### 2.4 Histopathologic analysis

Samples for histopathologic evaluation were dehydrated using a Leica tissue processor (Leica Biosystems), embedded in paraffin, and multiple sections (approximately 4 μm) were obtained. The slides were then stained with hematoxylin and eosin, and intestinal sections (cecum and colon) were scored by a board‐certified pathologist (Valerie Boras, MD) who was blinded to treatment. Total histopathologic scores from the cecum and colon were combined for a total possible score of 76. The scoring criteria included cell infiltrate severity (0–4), inflammation extent (0–3), epithelial hyperplasia (0–5), epithelial injury (0–4), cryptitis (2, 3), goblet cell loss (0–4), granulation tissue (4, 5), crypt loss (0–2), submucosal edema (0–3), and ulceration (3–5).

### 2.5 Quantification of inflammatory gene mRNA

To quantify mRNA targets of interest, RNA was extracted from 25 mg of cecum, proximal colon, and distal colon using a RNeasy Plus mini kit (Qiagen Inc., Germantown, MD, USA) following manufacturer's protocol. RNA quantity and quality were determined using a Bioanalyzer 2100 (Agilent Technologies Canada Inc., Mississauga, ON, Canada). RNA (1000 ng) was transcribed into complementary DNA (cDNA) using high‐capacity cDNA reverse transcription (Applied Biosystems, Waltham, MA, USA). Expression of mRNA for interferon‐gamma (Ifnγ), interleukin 1a (Il1a), Il1b, Il10, Il17a, and tumor necrosis factor‐alpha (Tnfα) were standardized against hypoxanthine‐guanine phosphoribosyltransferase (Hprt), β‐actin (Actb), and peptidylprolyl isomerase A (Ppia) using qBase+ software (Biogazelle, Gent, Belgium). These housekeeping genes were selected due to their low variation among samples. Quantitative polymerase chain reaction (PCR) reactions were run in a QuantStudio 5 thermocycler (Thermo Fisher Scientific Inc.). Each reaction contained 5 μL of PerfeCTa SYBR Green SuperMix (Quantabio, Beverly, MA, USA), 0.5 μL of each primer (10 μmol/L), 3 μL of RNase‐free water, and 1 μL of cDNA. Cycle conditions were 95°C for 15 min, 40 cycles of 95°C for 15 s, 55–60°C for 30 s, and 72°C for 30 s. A melt curve analysis was included in each run (55–95°C). Each reaction was run in triplicate, and the mean of the three observations was used for analysis.

### 2.6 Analyses

Statistical analyses were performed using Statistical Analysis Software (SAS Institute Inc. Cary, NC, USA). For continuous data (i.e., colon length and gene expression), the mixed procedure of SAS was used. In conjunction with a significant main effect, the least square means test was used to compare treatments. For categorical data (i.e., histopathologic change scores), a pairwise Fisher's exact test was used.

## 3 RESULTS

### 3.1 Piroxicam administration impacted colon weight and length

**FIGURE 1:**
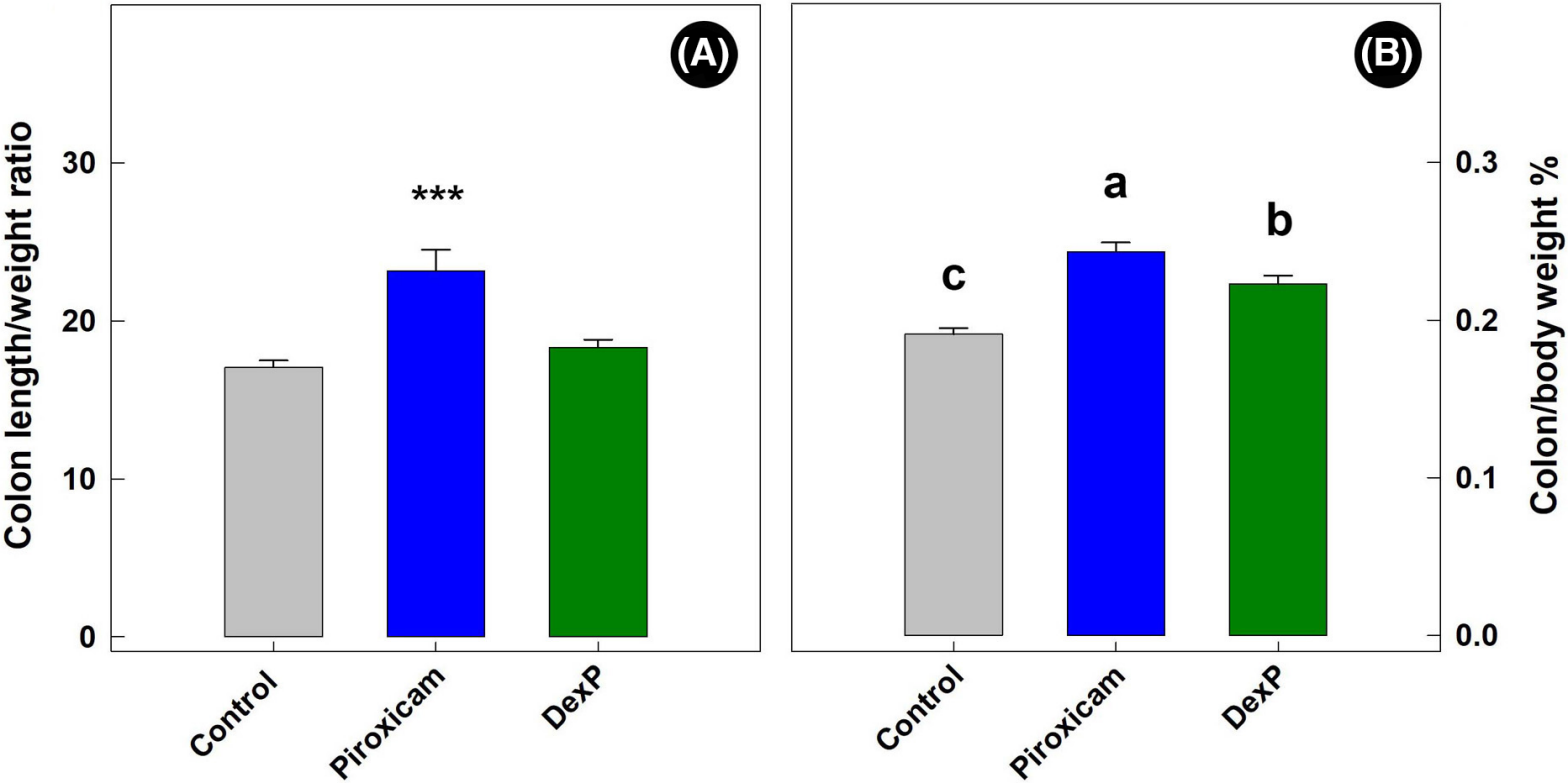
(A) Colonic length (in centimeters) to colonic weight (in grams) ratio in mice administered piroxicam (Piroxicam), mice administered piroxicam and dexamethasone (DexP), and mice not administered piroxicam or dexamethasone (Control). Asterisks (***) indicate that piroxicam treatment mice differed (*p* < 0.001). (B) Colonic weight (in grams) relative to body weight (in grams) (%) of mice administered piroxicam (Piroxicam), administered piroxicam and dexamethasone (DexP), or not administered piroxicam or dexamethasone (Control). Histogram bars denoted with a different letter (“a,” “b,” and “c”) differ (*p* ≤ 0.033). Lines associated with histogram bars represent standard errors of the mean (*n* = 6).

TCRα‐deficient mice administered piroxicam presented a higher (*p <* 0.002) colon length‐to‐weight ratio than control and DexP treatment mice (Figure 1A). Moreover, the weight of colons (i.e., adjusted to body weight) of piroxicam treatment mice was higher than control (*p <* 0.001) and DexP (*p <* 0.033) treatment mice (Figure 1B). The colons of DexP treatment mice also weighed more (*p =* 0.001) than colons of control treatment mice.

### 3.2 Inflammation was more pronounced in the proximal colon

**FIGURE 2:**
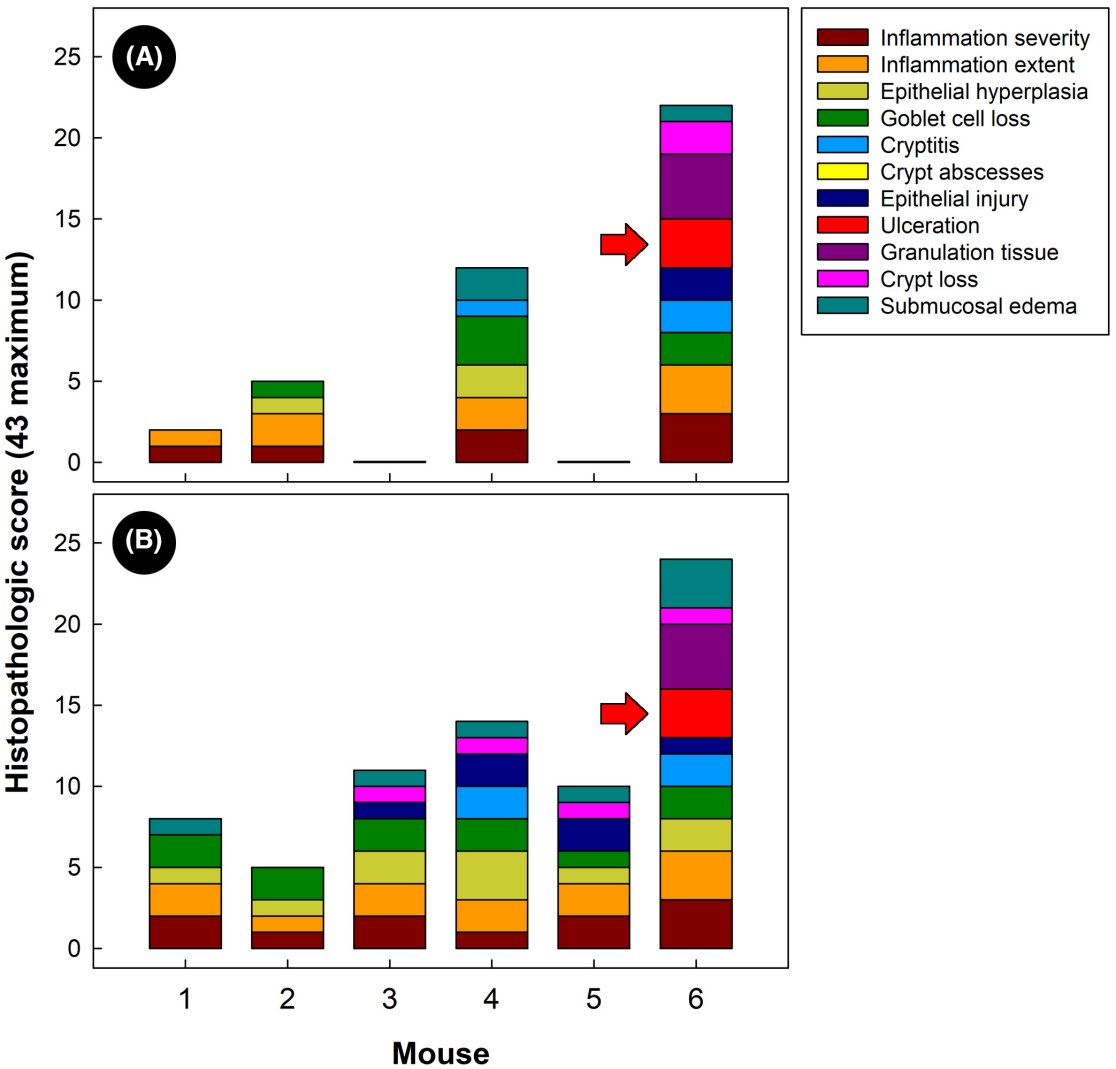
Total histopathological score and scores for individual criteria (stacked histogram) for the six mice administered piroxicam. (A) Cecum. (B) Proximal colon. Arrows point to ulceration that occurred in the cecum and proximal colon of mouse #6.

**FIGURE 3:**
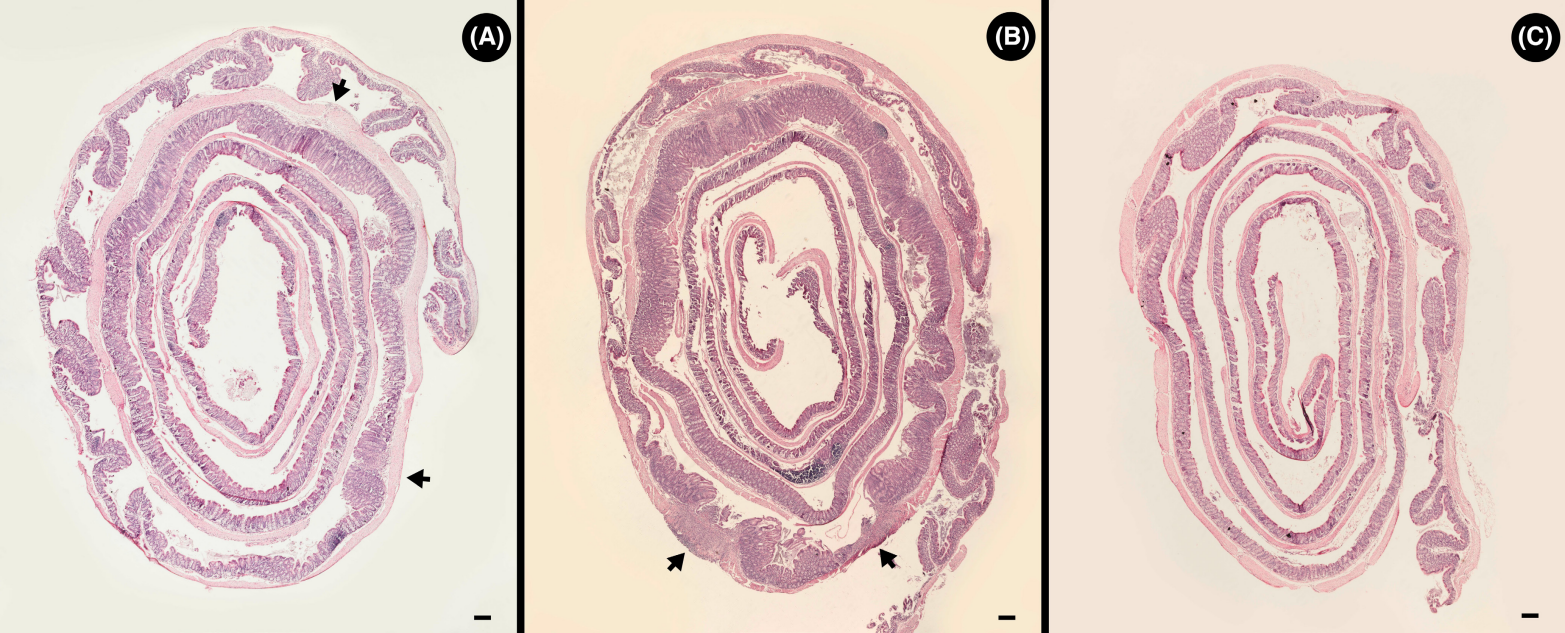
Representative hematoxylin and eosin (H&E)‐stained Swiss roll images that show multifocal diffuse inflammation in the colon. The proximal colon is rolled in the outer 2–3 layers transitioning into the distal colon located in the inner rolled section. (A) Mouse administered piroxicam. Arrows denote the interruption of healthy sections by diffuse focal areas of inflammation. The distal colon presents no inflammation. (B) Mouse administered piroxicam, showing extensive areas of inflammation and tissue damage throughout the proximal colon. Arrows indicate ulceration. (C) Mouse not administered piroxicam (Control) exhibiting a healthy colon. Scale bar = 1 mm.

**FIGURE 4:**
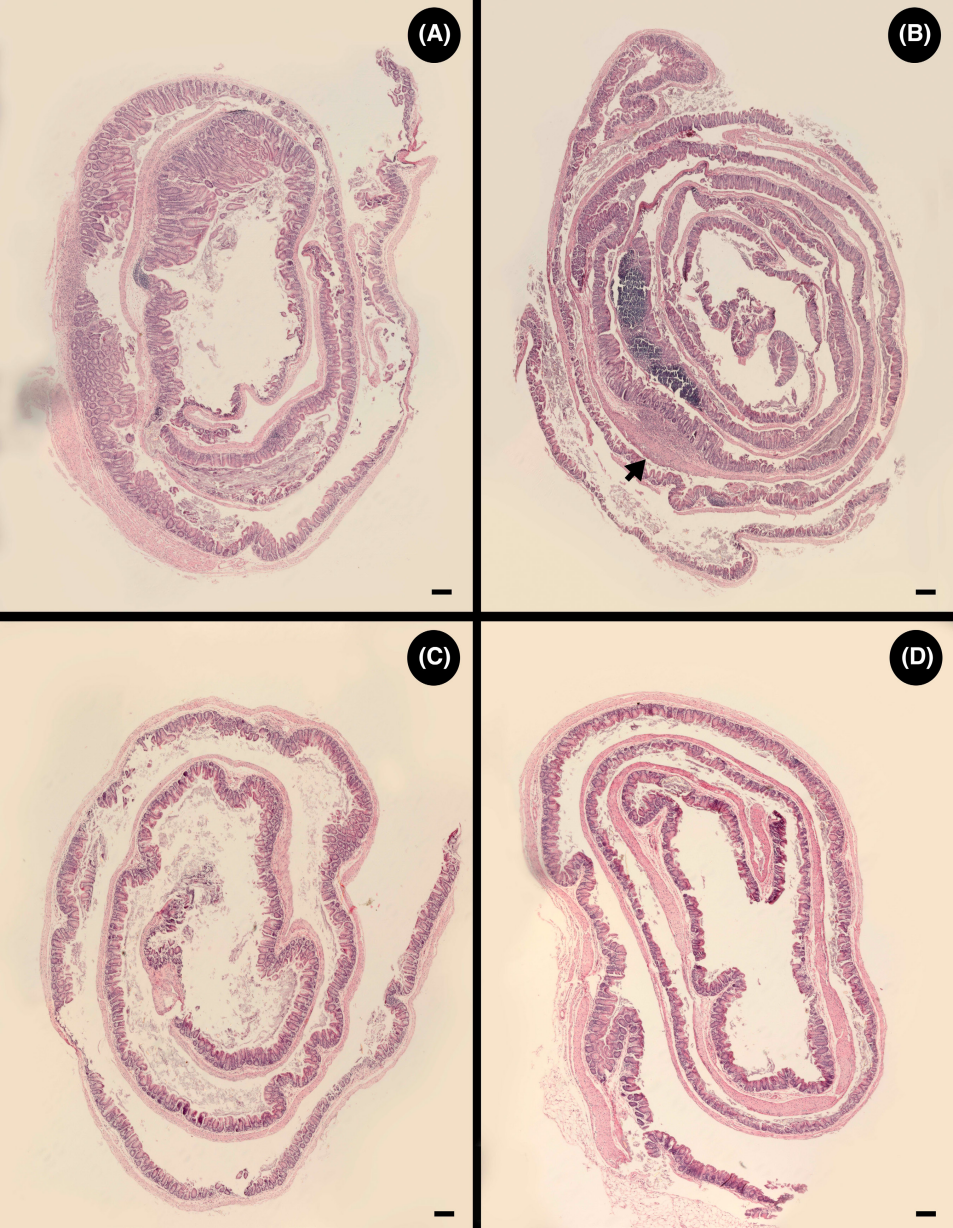
Representative hematoxylin and eosin (H&E) stained Swiss roll images that show multifocal diffuse inflammation in the cecum. (A) Mouse administered piroxicam showing extensive areas of inflammation and tissue damage throughout the cecum. (B) Mouse administered piroxicam showing extensive non‐inflamed areas except for a large zone of extensive infiltration and tissue damage with consequent ulceration (arrow). (C) Mouse administered piroxicam showing no inflammation in the cecum. (D) Mouse not administered piroxicam (Control) showing a healthy cecum. Scale bar = 1 mm.

Higher (*p* = 0.001) overall histopathological scores were observed in the proximal colon than in the cecum of piroxicam treatment TCRα‐deficient mice (Figure 2). However, variation in scores occurred among replicate animals, and two of the ceca of piroxicam treatment mice did not present inflammation and were scored as zero for every histopathological parameter. No histopathologic changes were observed in the distal colon of TCRα‐deficient mice administered piroxicam, and inflammation was restricted to the proximal colon, as well as the cecum (Figures 3 and 4).

### 3.3 Multifocal segmental inflammation occurred in the cecum and colon

In TCRα‐deficient mice administered piroxicam, inflammation in the proximal colon was localized in single or multiple focal points of infiltration with focused tissue damage (Figure 3A). Large areas of healthy tissue were observed in between focal points of inflammation. Variability was observed among replicate mice with only one individual presenting a uniform widespread inflammation in the proximal colon that progressed into ulceration (Figure 3B). Control treatment mice exhibited no inflammation in the proximal colon (Figure 3C). Piroxicam administration also resulted in areas of single or multiple focal points of infiltration within the cecum (Figure 4A,B), but not all animals administered piroxicam exhibited evidence of inflammation in the cecum (Figure 4C). All control treatment mice presented a healthy cecum (Figure 4D).

### 3.4 The severity of inflammatory cell infiltrate was variable in mice administered piroxicam

**FIGURE 5:**
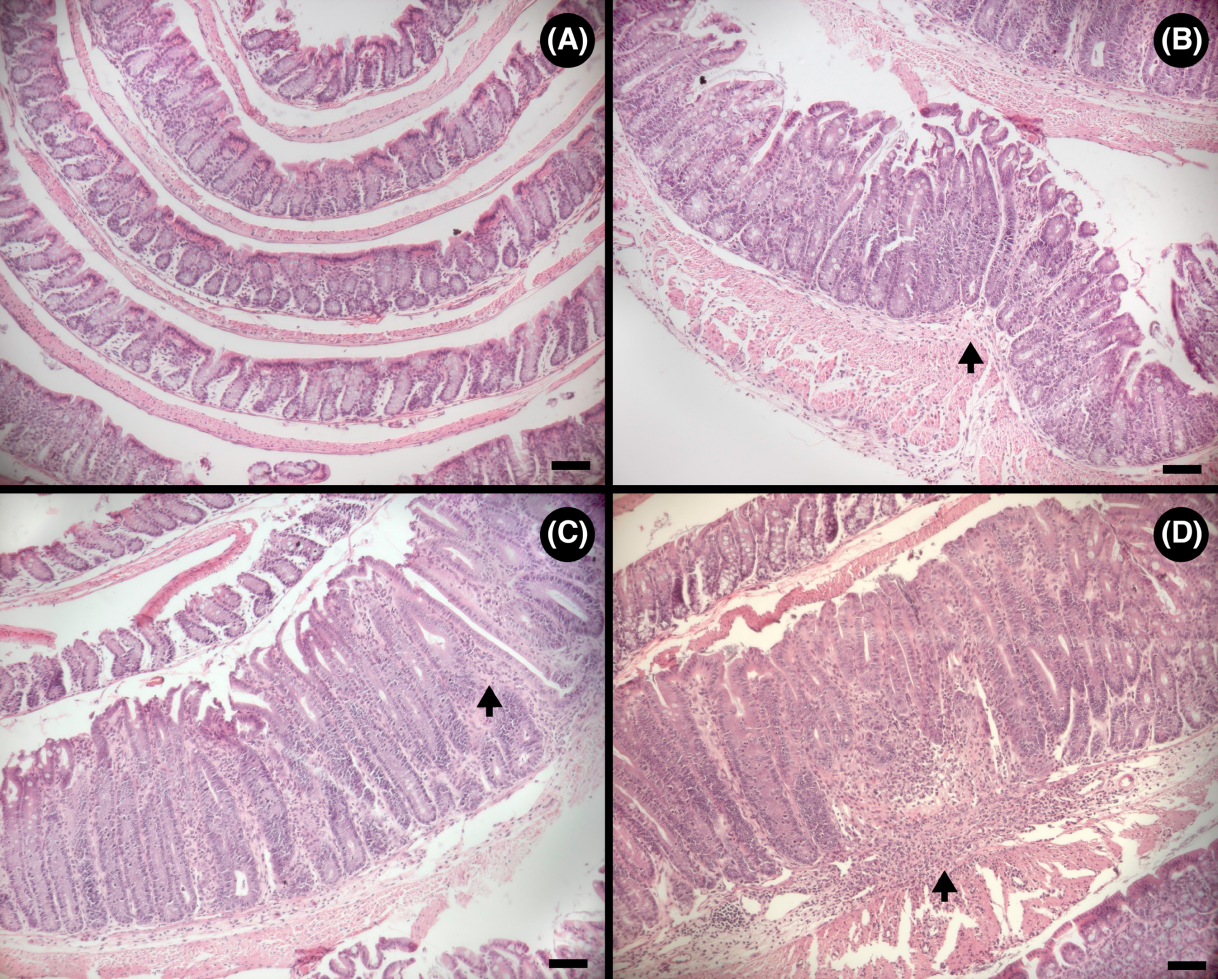
Representative hematoxylin and eosin (H&E)‐stained Swiss roll images that show the varying degrees of histopathologic changes in the proximal colon (infiltrate severity and infiltrate extent). (A) Mouse not administered piroxicam (Control) showing a healthy proximal colon. (B) Mouse administered piroxicam showing minimal infiltrate severity and infiltrate extent (arrow) (score of 1). (C) Mouse administered piroxicam showing mild infiltrate severity and infiltrate extent (arrow) (score of 2). (D) Mouse administered piroxicam showing moderate infiltrate severity and infiltrate extent (arrow) (score of 3). Scale bar = 1 mm.

Fourteen days after commencement of piroxicam administration, the severity and extent of leucocyte infiltrate in the proximal colon varied among individual TCRα‐deficient mice (Figure 5). In this regard, we observed that the infiltrate severity ranged from low to moderate (i.e., a score of 1 to 3, respectively). No cell infiltration was observed in control treatment mice (Figure 5A). In piroxicam treatment mice, the extent of cell infiltration ranged from shallow mucosal to transmural (Figure 5B–D), and occasionally ulceration. In the cecum of piroxicam treatment mice, a range of infiltrate severity ranging from absent to moderate was observed (Figure S1A–D). Similar to the proximal colon, the extent of leucocyte infiltration in the cecum of piroxicam treatment mice was variable, ranging from none (Figure S1B) to connective tissue infiltration (Figure S1C), and in more severe cases, transmural infiltration (Figure S1D).

### 3.5 Piroxicam treatment stimulated marked epithelial hyperplasia and goblet cell loss

**FIGURE 6:**
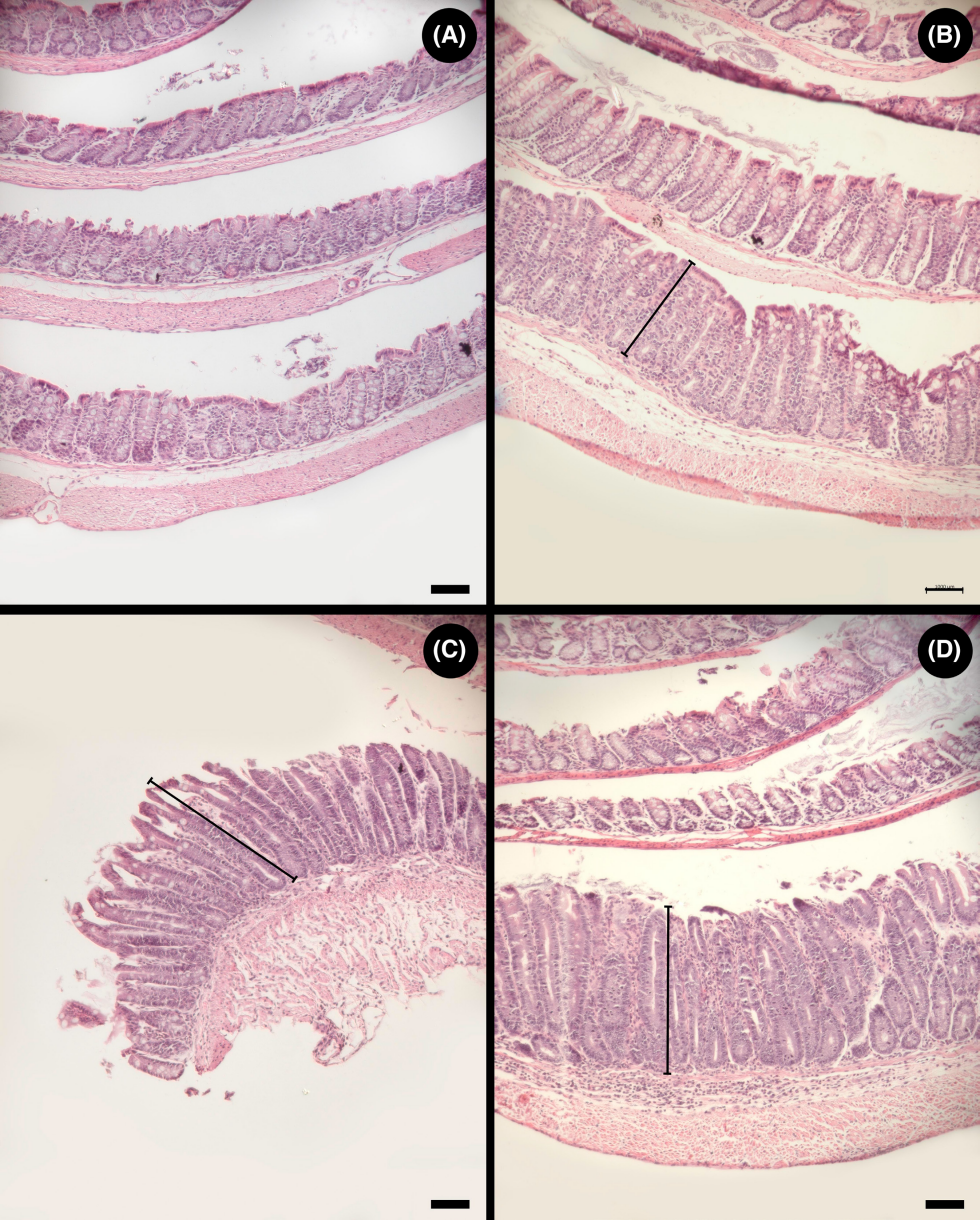
Representative hematoxylin and eosin (H&E)‐stained Swiss roll images that show the varying degrees of histopathologic changes in the proximal colon (epithelial hyperplasia and goblet cell loss). (A) Mouse not administered piroxicam (Control) showing a healthy proximal colon. (B) Mouse administered piroxicam showing minimal epithelial hyperplasia and goblet cell loss (line) (score of 1). (C) Mouse administered piroxicam showing mild epithelial hyperplasia and goblet cell loss (line) (score of 2). (D) Mouse administered piroxicam showing moderate epithelial hyperplasia and goblet cell loss (line) (score of 3). Scale bar = 1 mm.

**FIGURE 7:**
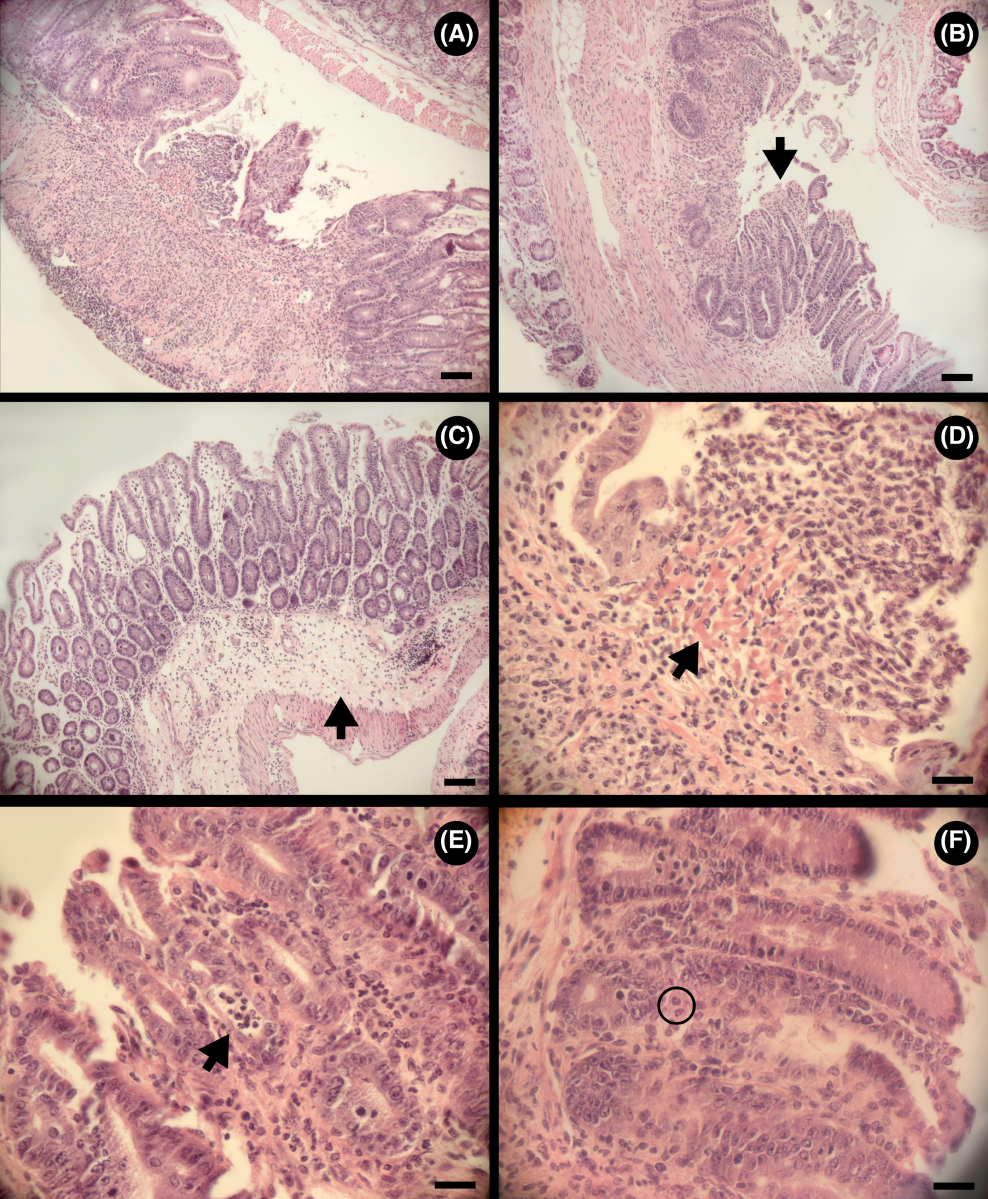
Representative hematoxylin and eosin (H&E)‐stained Swiss roll images that show varying histological features found in mice administered piroxicam. (A) Ulceration in the proximal colon (scale bar = 1 mm). (B) Epithelial injury (arrow) in the proximal colon (scale bar = 1 mm). (C) Prominent edema (arrow) in the submucosa of the cecum (scale bar = 1 mm). (D) Presence of granulation tissue in an ulcer in the proximal colon (arrow) (scale bar = 100 μm). (E) Crypt abscess in the proximal colon showing the presence of neutrophils in the lumen of the crypt (arrow) (scale bar = 100 μm). (F) Cryptitis and neutrophils (circle) in between crypts in the proximal colon (scale bar = 100 μm).

Conspicuous epithelial hyperplasia and goblet cell loss were observed in the colons of TCRα‐deficient mice administered piroxicam (Figure 6). However, the degree of injury ranged from minimal to mild (Figure 6A–D). Although less pronounced relative to the colon, epithelial hyperplasia and goblet cell loss also occurred in the cecum (Figure 7C).

### 3.6 Additional histopathologic changes were observed in mice administered piroxicam

**FIGURE 8:**
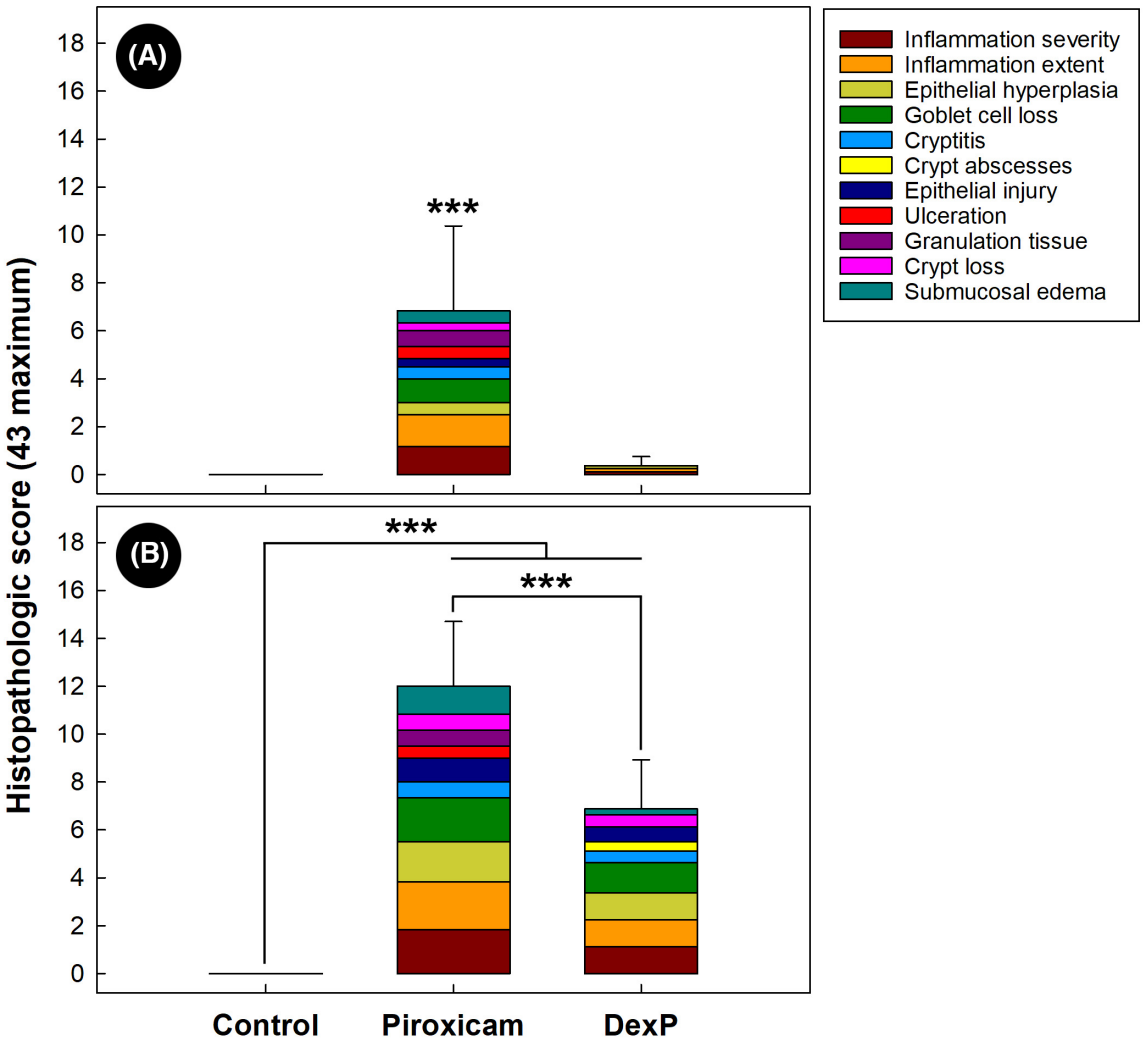
Average total histopathologic scores and average scores for individual criteria (stacked histogram) for mice administered piroxicam (Piroxicam), mice administered piroxicam and dexamethasone (DexP), and mice not administered piroxicam or dexamethasone (Control). (A) Cecum. (B) Proximal colon. Histogram bars denoted with *** differ (*p* < 0.001). Lines associated with histogram bars represent standard errors of the mean (*n* = 6).

Ulceration (Figure 7A), epithelial injury (Figure 7B), submucosal edema (Figure 7C), granulation tissue (Figure 7D), crypt abscesses (Figure 7E), and cryptitis (Figure 7F) were observed in TCRα‐deficient mice administered piroxicam. Additional histopathologic changes were scored individually as well (Figure 8).

### 3.7 Administration of dexamethasone ameliorated intestinal inflammation

The total histopathologic scores were lower (*p <* 0.001) in the cecum and proximal colon of TCRα‐deficient mice administered piroxicam and dexamethasone compared to mice administered piroxicam alone (Figure 8A,B). Notably, all individual scoring parameters were reduced in DexP treatment mice. However, inter‐animal variation was observed, and some individuals presented no histopathologic changes (17%), whereas others presented mild‐to‐moderate inflammation (83%) (Figure S2), including conspicuous epithelial hyperplasia, goblet cell loss, cryptitis, and crypt abscesses. There were no observable differences in histopathologic change scores between control and DexP treatment mice in the cecum. However, control treatment mice had significantly lower scores than DexP treatment mice in the colon (*p <* 0.001).

### 3.8 Administration of dexamethasone reduced expression of inflammation markers

**FIGURE 9:**
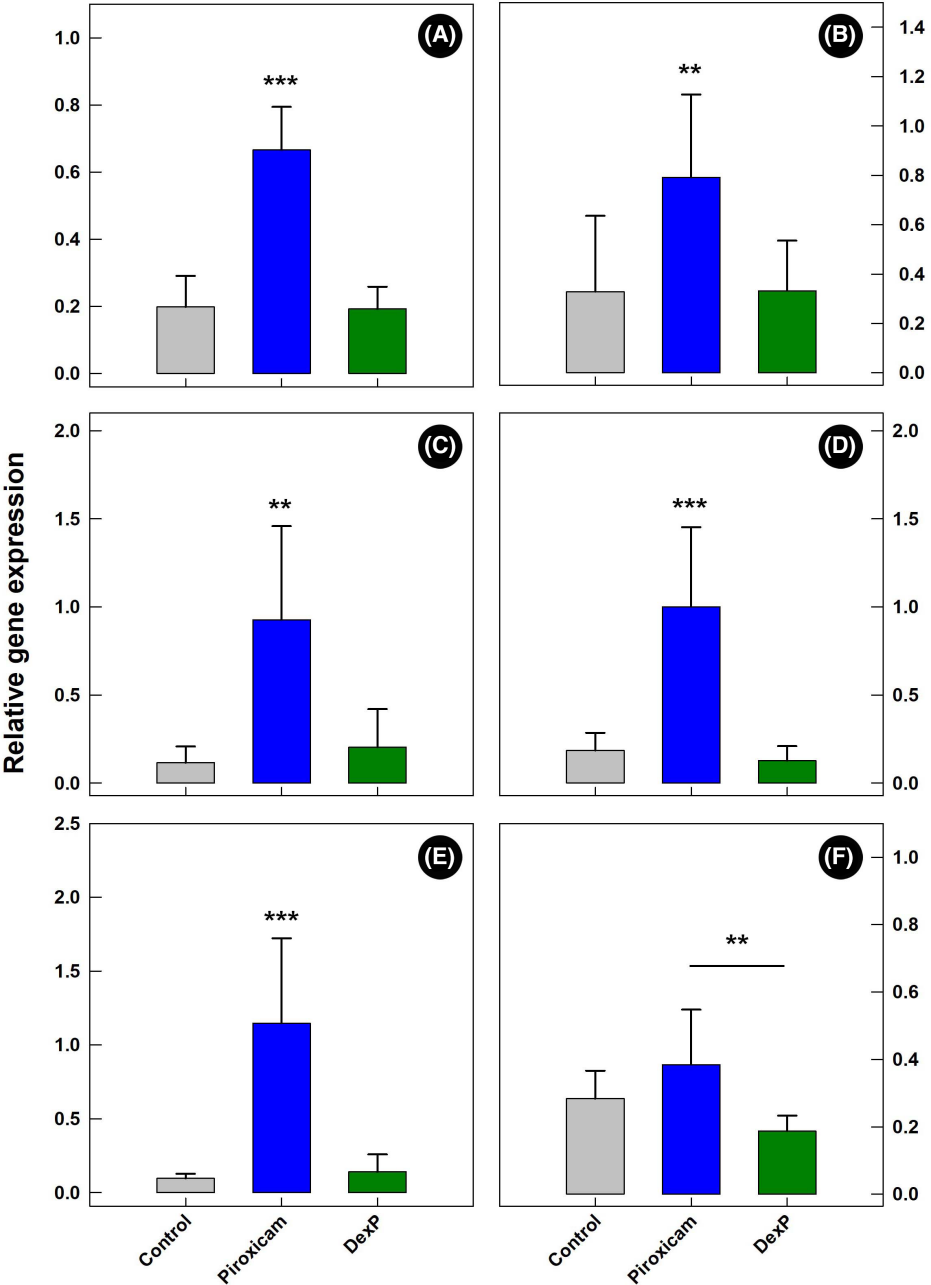
Relative expression of messenger RNA (mRNA) in the proximal colon of mice administered piroxicam (Piroxicam), mice administered piroxicam and dexamethasone (DexP), and mice not administered piroxicam or dexamethasone (Control). (A) Interferon‐gamma (Ifnγ). (B) Tumor necrosis factor‐alpha (Tnfα). (C) Interleukin 1a (Il1a). (D) Il1b. (E) Il17a. (F) Il10. Lines associated with histogram bars represent standard errors of the mean (*n* = 6). Histogram bars denoted with ** differ (*p* ≤ 0.010). Histogram bars denoted with *** differ (*p* ≤ 0.001).

Higher quantities (*p ≤* 0.025) of Ifnγ, Tnfα, and Il1a were observed in the cecum of TCRα‐deficient mice administered piroxicam alone in comparison to mice administered piroxicam and dexamethasone (Figure S3). Moreover, higher (*p ≤* 0.019) quantities of Ifnγ, Tnfα, Il1a, Il1b, and Il17a mRNA were observed in the proximal colon of piroxicam treatment mice relative to control and DexP treatment mice (Figure 9A–E). Differences in quantities of Il10 mRNA (*p =* 0.026) were observed between piroxicam and DexP treatment mice in the proximal colon (Figure 9F).

## 4 DISCUSSION

TCRα‐deficient mice are used as a spontaneous colitis model, including as a model of ulcerative colitis.^5^ Bhan et al.^13^ showed a temporal progress of colonic disease in TCRα‐deficient mice with 60%, 83%, and 93% of the mice exhibiting disease by the age of 20–25, 25–30, and >30 weeks, respectively. The late and uneven temporal onset of colonic disease among individual animals represents a logistical challenge for conducting experiments involving time‐targeted treatments. To synchronize disease onset, Nishiyori et al.^11^ provided piroxicam in feed; piroxicam has been previously used as a colitis incitant in an IL10^−/−^ (IL10‐deficent) murine model^14^ in which spontaneous colitis developed after a 14‐day treatment in 4‐ to 6‐week‐old mice. The administration of piroxicam induces intestinal apoptosis in vivo and in vitro (i.e., in CT26 murine colon carcinoma cells).^14^ Although the exact mechanisms by which piroxicam induces apoptosis are not known, it is believed to be related to the inhibition of prostaglandin production.^14^ It is hypothesized that apoptosis facilitates enteric bacterial contact with the epithelium, thus elevating the intestinal response to the microbiota.^14^ As IL10‐deficient mice possess a reduced ability to regulate the inflammatory response, the result is an acceleration of colitis development. It is plausible that a similar mechanism of action occurs in TCRα‐deficient mice administered piroxicam. It is noteworthy that neither the IL10‐deficient nor TCRα‐deficient mice develop colitis under germ‐free conditions, which implicates the microbiota in colitis development.^15^

The salient advantage to piroxicam‐triggered colitis in TCRα‐deficient mice is that a high prevalence of young animals will develop colitis simultaneously. Similarly to Nishiyori et al.,^11^ we observed that the daily administration of piroxicam for 14 days to 6‐week‐old TCRα‐deficient mice resulted in all of the mice developing colitis. The TCRα‐deficient mice utilized in our experiment were bred and raised in a specific‐pathogen‐free (SPF) facility. Subsequently, they were maintained in IVCs on sterilized food and water during the experimental period. TCRα‐deficient mice raised in a conventional facility exhibited a reduced prevalance of colitis as compared to mice maintained in an SPF facility.^16^ This is consistent with the “hygiene hypothesis” in which a reduced exposure to microorganisms at an early stage of life will lead to Th2‐mediated allergic diseases and inflammatory bowel disease.^16^ As noted earlier, a diverse intestinal microbiota is essential for the development of colitis, given that TCRα‐deficient mice do not develop colitis under germ‐free or specific gnotobiotic conditions.^17^, ^18^ The bacteria responsible for initiating colitis in this model are still unknown, and this warrants further investigation.

Inflammation triggered by piroxicam in young TCRα‐deficient mice differs from inflammation that develops spontaneously in older TCRα‐deficient mice. Rectal lesions are prominent in spontaneous inflammation.^5^ After the administration of piroxicam to TCRα‐deficient mice, we observed that lesions developed in the proximal colon, and to a lesser extent in the cecum, but no evidence of pathologic changes was observed in the rectum. Continuous marked dilation and thickening of the cecum and colon were observed in TCRα‐deficient mice developing spontaneous disease.^5^ In contrast, we observed that piroxicam‐induced disease was most often multifocal with segmental areas of infiltration and tissue damage separated by healthy segments of intestine. Extensive tissue damage and inflammation that affected most of the proximal colon were observed in only a subset of mice. It is important to highlight that we utilized a Swiss roll technique to histologically observe the entirety of the colon and cecum. Given the multifocal segmental nature of disease, it is easy to miss the site of inflammation if only a small section of the intestine is analyzed. In contrast to Nishiyori et al.,^11^ we did not observe any signs of leukocytic infiltration, mucosal hyperplasia, or goblet cell loss in the distal colon of TCRα‐deficient mice administered piroxicam. It is noteworthy that Nishiyori et al.^11^ did not report disease in the cecum nor the multifocal segmental nature of the inflammation.

We observed that the administration of piroxicam to TCRα‐deficient mice resulted in a higher colon length‐to‐weight ratio than in TCRα‐deficient mice not administered piroxicam. A higher presence of inflammatory cells (lymphocytes, macrophages, and plasma cells), as well as hyperplasia of the epithelium and edema, will result in thickening of the colonic tissue.^19^

Mucosal infiltration, epithelial hyperplasia, and goblet cell loss were the dominating histopathologic features observed in TCRα‐deficient mice administered piroxicam, which is consistent with previous findings.^13^ A striking histological feature that we observed was the presence of ulceration, both in the proximal colon and cecum. Ulcers do not develop in the spontaneous disease in TCRα‐deficient mice.^5^, ^13^, ^17^ We observed that ulcers developed only in one out of six of TCRα‐deficient mice administered piroxicam. Moreover, considerable variation in histopathologic changes was observed among individuals (primarily in inflammation severity, leukocyte infiltrate extent, epithelial hyperplasia, and goblet cell loss), which was not originally described by Nishiyori et al.^11^ The reasons for the variation in disease observed among TCRα‐deficient mice administered piroxicam are unknown, but this variation has implications for using this model. It is possible that individual mice may have differential susceptibilities to the effects of piroxicam on the intestinal tissue. It is also possible that variability within replicates could be associated with a different time of the onset of inflammation. Suceptibility to piroxicam could have incited inflammation at earlier stages in some mice, wheras others developed it later during the 14 days of treatment. Commensal bacteria are involved in the onset and severity of inflammation.^17^ Even though the structure of the enteric microbiota in mice housed within our animal facility is quite uniform (data not shown), it is plausible that variations in the inflammatory damage could partly be due to slight variations in the composition of the bacterial community, and this warrants investigation.

We examined the impact of the anti‐inflammatory drug dexamethasone that was administered daily for 14 days, on inflammation in TCRα‐deficient mice administered piroxicam. In contrast to Nishiyori et al.,^11^ colonic inflammation was not resolved in mice administered dexamethasone. Variable impacts of dexamethasone among replicate mice were observed, ranging from complete amelioration in some mice to no amelioration in others. Contrary to the outcome in the colon, inflammation was consistently reduced by dexamethasone in the cecum. Expression of pro‐inflammatory cytokines Ifnγ, Tnfα, Il1a, Il1b, and Il17a mRNA was elevated in the proximal colon of piroxicam TCRα‐deficient mice not administered dexamethasone. This is consistent with the observed histopathologic changes that were accompanied by upregulation of the expression of pro‐inflammatory markers, such as TNFα, that promote vasodilation benefiting the influx of inflammatory mediators and leukocytes to the affected area.^20^ Furthermore, TNFα will promote angiogenesis and intraepithelial cell damage, induce Paneth cell death, and activate both macrophages and effector T cells in animals with colitis.^21^ Therefore, many experimental treatments targeting colitis in mice aim to inhibit TNF receptors.^22^, ^23^ Similar to Nishiyori et al.,^11^ we observed that the expression of Ifnγ was lower in the proximal colon of mice administered piroxicam and dexamethasone. We also observed a decrease in Il1a and Il1b mRNA in mice administered piroxicam and dexamethasone. In the spontaneous TCRα‐deficient model of colitis, an increase in IL1A and IL1B occurs in young mice (age 4–8 weeks), suggesting that the lack of TCR receptor will consequently lead to an increase in the expression of these cytokines even before there is histological evidence of colitis.^19^ Moreover, these cytokines remain elevated in mice beyond the age of 20 weeks. IL1 is thought to play an essential role in the commencement of colonic inflammation.^21^ We observed that Il17a mRNA was elevated in TCRα‐deficient mice administered piroxicam, and that the administration of dexamethasone reduced Il17a levels to baseline. Increased expression of IL17a was also observed in the spontaneous TCRα‐deficient model of colitis.^6^ IL17‐producing cells are concentrated in the intestinal mucosa and submucosa of patients with inflammatory bowel disease, and they are functionally linked with a pro‐inflammatory role via the upregulation of TNFα, IL1B, IL6, IL8, and neutrophil recruitment.^21^, ^24^ Induction of colitis with trinitrobenzenesulfonic acid in IL17R knockout mice demonstrated the involvement of IL17 in preventing weight loss, colonic inflammation, and IL6 production.^25^ IL10 plays an important role in the regulation of colitis,^26^, ^27^ and we observed a higher relative expression of Il10 in TCRα‐deficient mice administered piroxicam compared to mice administered piroxicam and dexamethasone; an elevated expression of IL10 is consistent with a host's attempt to regulate inflammation.

## 5 CONCLUSION

We completed an in‐depth histopathological analysis of piroxicam‐triggered colitis in TCRα‐deficient mice. This is a useful model to study cecal and colonic inflammation, and in particular, crypt hyperplasia, goblet cell loss, and ulceration. However, the colonic inflammation encountered using this model is limited to the proximal colon; it is multifocal, segmental, and spatially variable. Moreover, variation in the degree of colitis occurs among replicate animals. Thus, it is imperative that researchers analyze the proximal colon in its entirety and collect samples for analyses that reflect the variable nature of colitis. A future step to improve the model could focus on assessing the variability in when the inflammation is generated in each animal. This could be accomplished by sampling mice daily (within the 14‐day period of Piroxicam) to evaluate when the onset of disease develops. Additionally, analysis of bacterial communities could be conducted to evaluate how differences in microbiota composition can impact the development of the disease.

## AUTHOR CONTRIBUTIONS

Maximo E. Lange, Danisa M. Bescucci, and G. Douglas Inglis designed the study. Maximo E. Lange and Danisa M. Bescucci performed experiments. Valerie F. Boras scored tissues for histopathologic changes. Maximo E. Lange, Danisa M. Bescucci, and G. Douglas Inglis analyzed the data. Maximo E. Lange, G. Douglas Inglis and Tony Montina generated the initial draft of the manuscript. All authors reviewed and revised the manuscript. This study was supervised by G. Douglas Inglis.

## FUNDING INFORMATION

Funding was provided by AAFC (project 2991), the Canadian Glycomics Network (CD‐50), and Alberta Innovates (CD‐60).

## CONFLICT OF INTEREST STATEMENT

The authors declare no conflict of interest.

## ETHICS STATEMENT

All experiments involving mice were carried out in strict accordance with the recommendations specified in the Canadian Council on Animal Care Guidelines. The project was reviewed and approved by the AAFC LeRDC Animal Care Committee (Animal Use Protocol #2008).

## Supporting information


Figure S1.
Figure S2.
Figure S3.
