# Supplementary material for: Assessment of the piroxicam‐incited model of synchronized colitis in T‐cell receptor alpha chain‐deficient mice
Source: Animal Model Exp Med. 2024 Jul 11;7(4):533–43. doi: 10.1002/ame2.12456 (PMC11369028; doi:10.1002/ame2.12456)
Supplement: Supplementary file 1 — Figure S1. Figure S2. Figure S3. [file AME2-7-533-s001.docx]

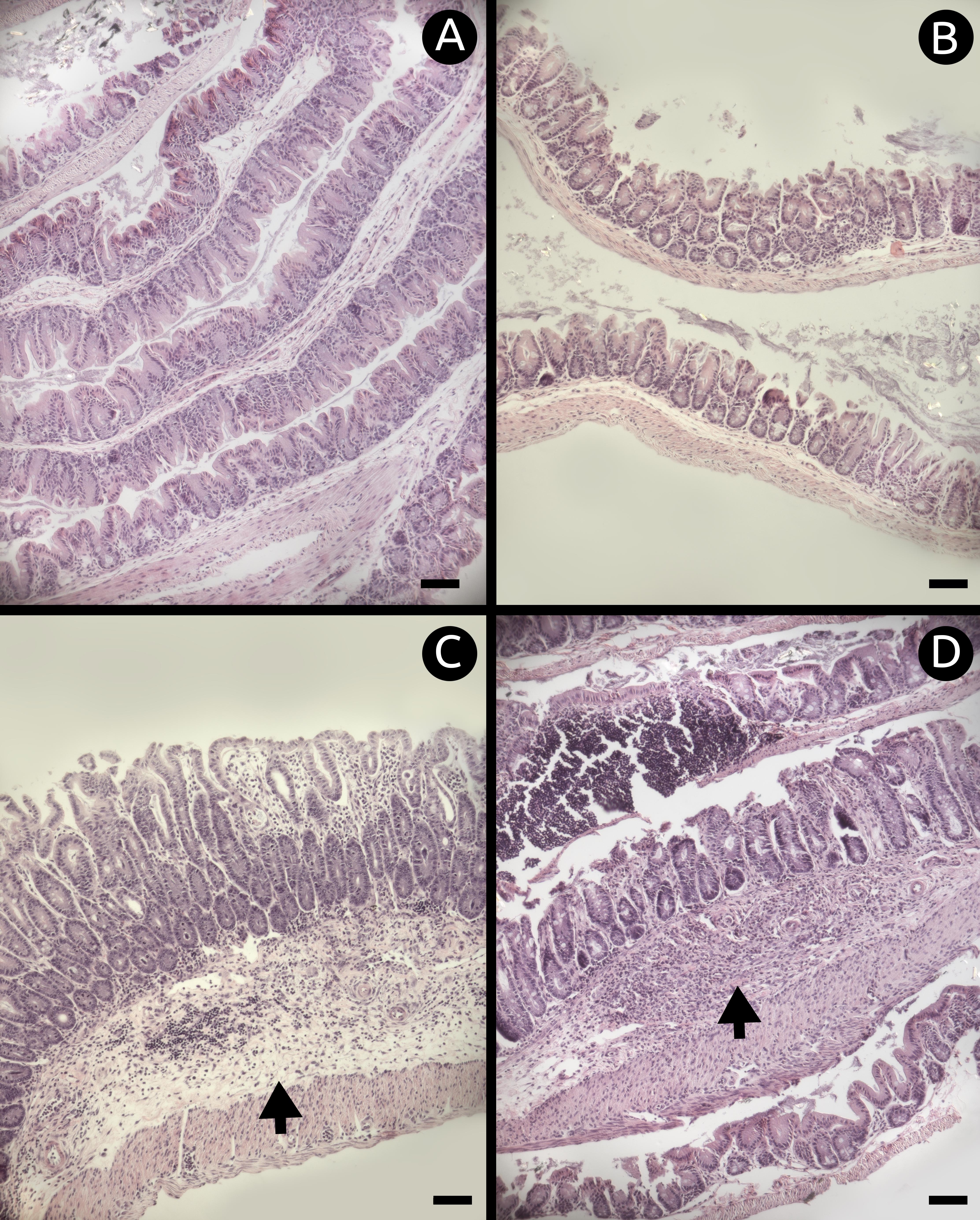


FIGURE S1 Representative images of H&E stained sections of the cecum depicting the varying degrees of infiltrate severity and infiltrate extent in mice administered piroxicam and in mice not administered piroxicam (Control). (A) Control mouse showing a healthy cecum. (B) Piroxicam treatment mouse presenting no inflammatory infiltrate. (C) Piroxicam treatment mouse with a score of 2 for infiltrate severity and extent. The arrow denotes edema in the submucosa. (D) Piroxicam treatment mouse with a score of 3 for infiltrate severity. The arrow denotes the extent with presence of fibrin in the submucosa. Scale bar=1 mm.

**
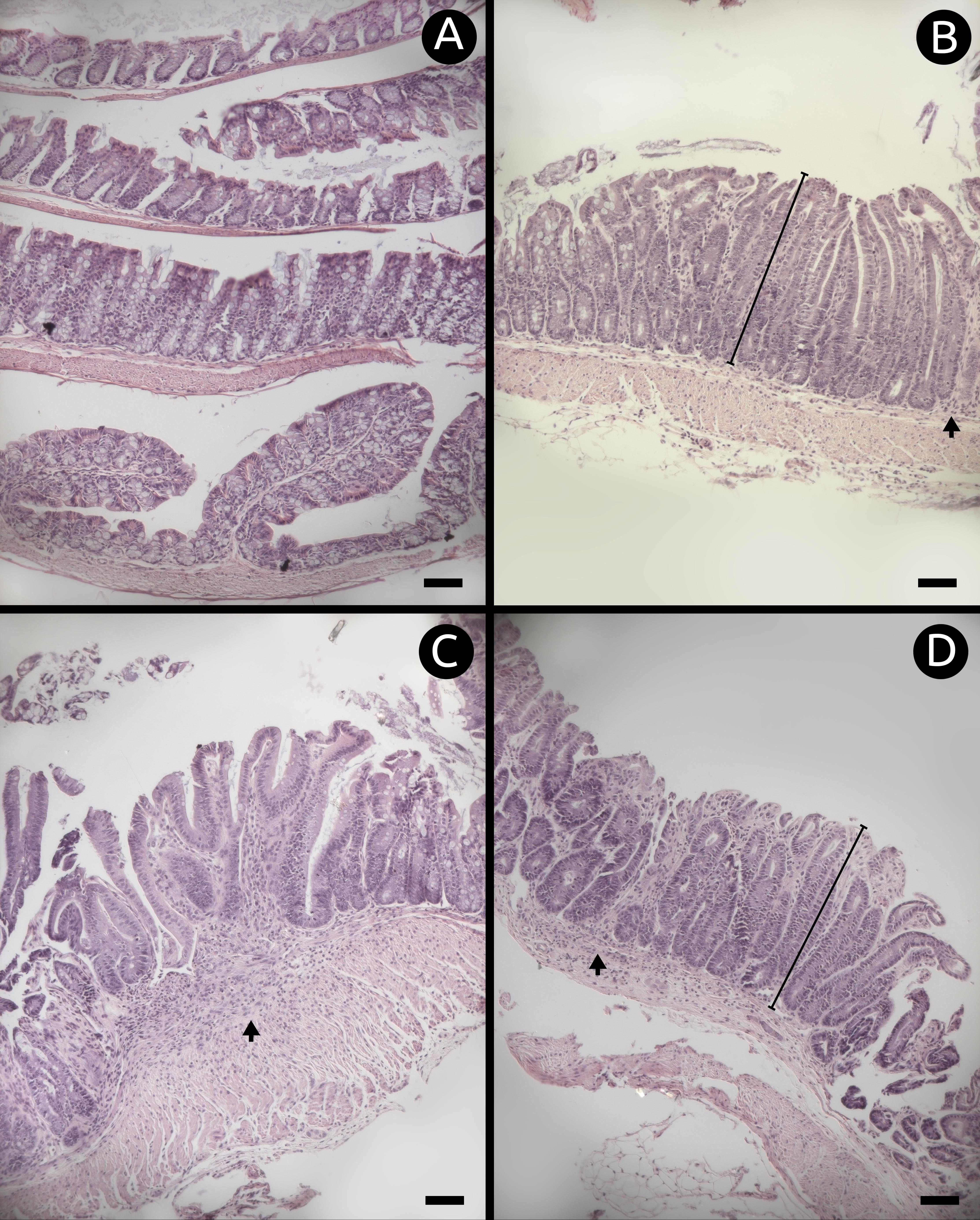
**

FIGURE S2 Representative images of H&E stained sections of proximal colon and cecum illustrating the varying degrees of inflammation and tissue damage observed in mice administered piroxicam and dexamethasone (DexP). (A) Proximal colon of a DexP mouse depicting healthy tissue. (B) Proximal colon of DexP treatment mouse with a low infiltrate (arrow), epithelial hyperplasia and loss of goblet cells (line). (C) Proximal colon of a DexP treatment mouse with an elevated score of leukocyte infiltrate severity and extent (arrow). (D) Cecum of a DexP treatment mouse depicting infiltration of leukocytes (arrow), loss of goblet cells and epithelial hyperplasia (line). Scale bar=1 mm.


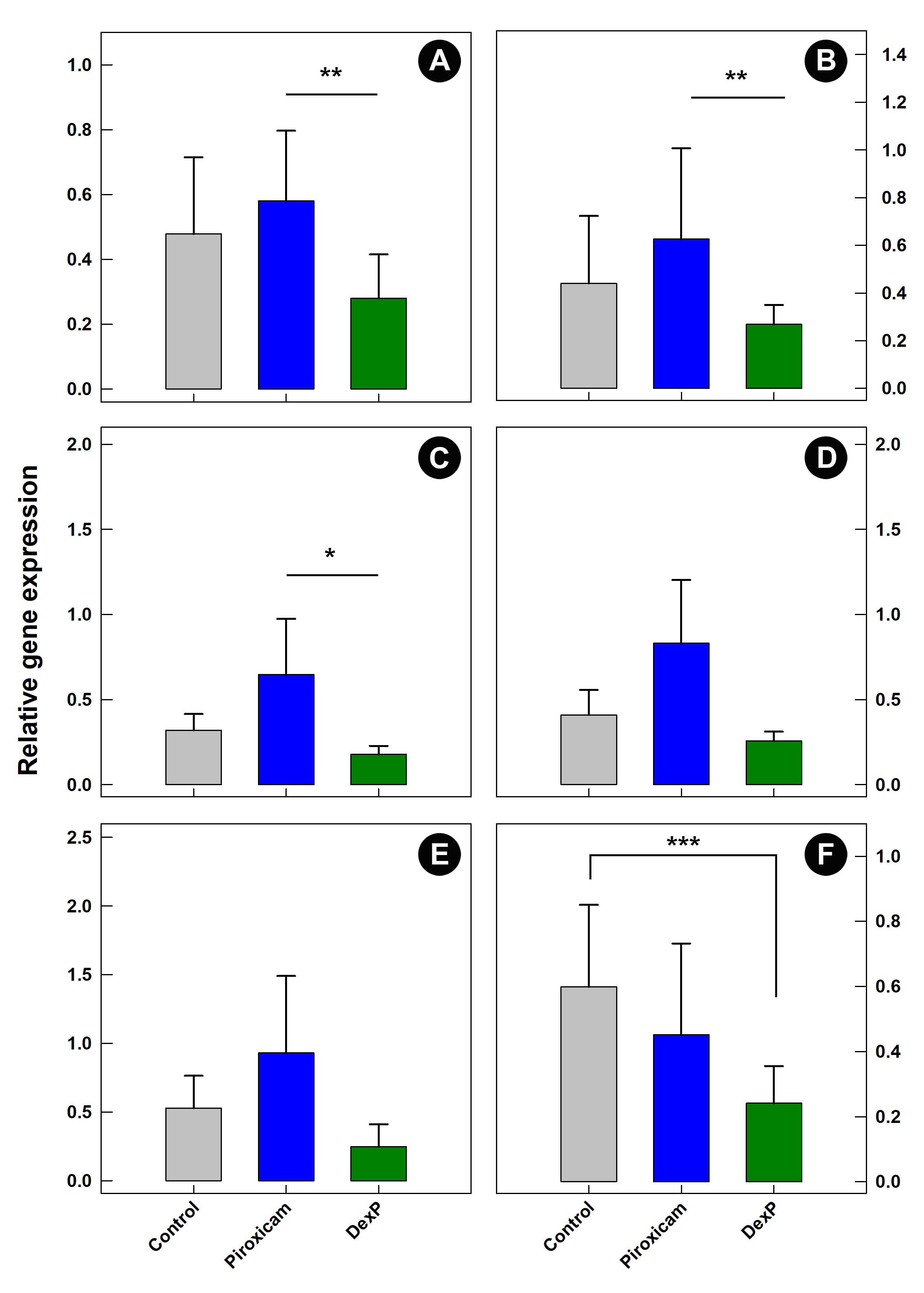


FIGURE S3 Relative expression of mRNA gene expression in the cecum of mice administered piroxicam (Piroxicam), mice administered piroxicam and dexamethasone (DexP), and mice not administered piroxicam or dexamethasone (Control). (A) *Ifnγ.* (B) *Tnfα.* (C) *Il1a.* (D) *Il1b.* (E) *Il17a.* (F) *Il10.* Lines associated with histogram bars represent standard errors of the mean (n=6). Histogram bars denoted with * differ (*p*≤0.050). Histogram bars denoted with ** differ (*p*≤0.010). Histogram bars denoted with *** differ (*p*<0.001).
